# Supplementary material for: Latent transforming growth factor binding protein-2 (LTBP2), an IPF biomarker of clinical decline, promotes TGF-beta signaling and lung fibrosis in mice
Source: bioRxiv. 2025 May 11:2025.05.06.652563. Preprint. [Version 1] doi: 10.1101/2025.05.06.652563 (PMC12247642; doi:10.1101/2025.05.06.652563)
Supplement: Supplementary file 1 [file media-1.pdf]

A

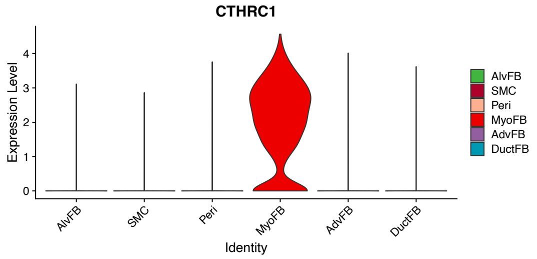

B

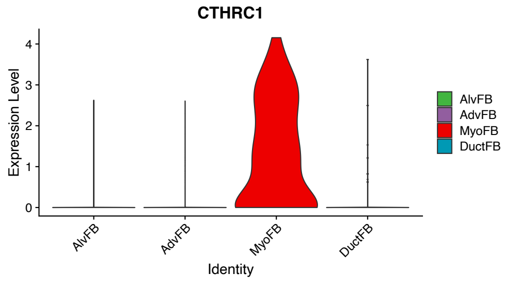

C

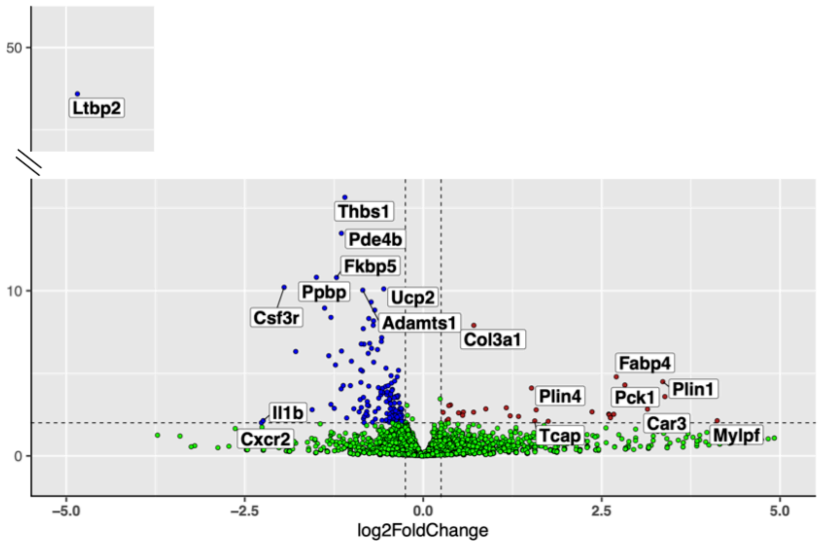

Figure S1. Cthrc1 expression in myofibroblasts (MyoFB) from human disease scRNASeq data.

A. Violin plot of *CTHRC1* expression in mesenchymal subsets from a human pulmonary fibrosis plus control dataset, Tsukui et al.

B. Violin plot of *CTHRC1* expression in fibroblast subsets from a human COPD plus control dataset.

C. Volcano plot of genes up and down-regulated in untreated LTBP2-/- versus control mouse lungs by bulk RNASeq.
